# Binding of Glycerol to Human Galectin-7 Expands Stability and Modulates Its Functions

**DOI:** 10.3390/ijms232012318

**Published:** 2022-10-14

**Authors:** Yebing Liang, Yuxiang Wang, Xingyu Zhu, Jun Cai, Anqi Shi, Jing Huang, Qiuju Zhu, Yunlong Si

**Affiliations:** Jiangsu Key Laboratory of Brain Disease and Bioinformation, Research Center for Biochemistry and Molecular Biology, Xuzhou Medical University, Xuzhou 221004, China

**Keywords:** galectin-7, glycerol, stability, crystal structure, aquaporin-3

## Abstract

Glycerol is seen in biological systems as an intermediate in lipid metabolism. In recent years, glycerol has been reported to act as a chemical chaperone to correct the conformation of proteins. Here, we investigate the role of glycerol in galectin-7 (Gal-7). The thermal shift and CD assays showed that the thermal stability of Gal-7 increased with glycerol concentration but with little secondary structure changes induced by glycerol. In addition, glycerol can inhibit Gal-7-mediated erythrocyte agglutination. We also solved the crystal structures of human Gal-7 in complex with glycerol in two different conditions. Glycerol binds at the carbohydrate-recognition binding sites of Gal-7, which indicates glycerol as a small ligand for Gal-7. Surprisingly, glycerol can bind a new pocket near the N-terminus of Gal-7, which can greatly reduce the flexibility and improve the stability of this region. Moreover, overexpression of Gal-7 decreased the intracellular triglyceride levels and increased mRNA expression of aquaporin-3 (AQP-3) when HeLa cells were incubated with glycerol. These findings indicate that Gal-7 might regulate glycerol metabolism. Overall, our results on human Gal-7 raise the perspective to systematically explore this so far unrecognized phenomenon for Gal-7 in glycerol metabolism.

## 1. Introduction

Galectins (Gals) are a family of animal lectins that bind β-galactose-containing glycoconjugates, and their carbohydrate recognition domain (CRD) has a conservative amino acid sequence. They are widely distributed in lower invertebrates to higher vertebrates, with a molecular weight between 14 and 35 kDa [1,2,3]. To date, 16 mammalian galectins have been discovered, and galectin-7 (Gal-7; also termed p53-induced gene 1 product), belonging to prototype galectins, is a multifunctional effector by productive pairing with distinct glycoconjugates and protein counter-receptors in the cytoplasm and nucleus, as well as on the cell surface [4].

Gal-7 participates in diverse processes, such as controlling apoptosis, cell migration and cell adhesion. In addition, it also plays a crucial role in the re-epithelialization process of corneal or epidermal wounds and in several human diseases/disorders, such as cancer [4,5]. Cervical cancer is the fourth most frequent cancer in women worldwide, representing nearly 8% of all female cancer deaths every year [6]. It is reported that Gal-7 is negatively regulated in cervical cancer. Increased Gal-7 expression has been shown as a positive predictive biomarker for clinical responses after adjuvant radiation therapy in cervical cancer patients [7]. Ectopic expression of Gal-7 in HeLa cells renders the cells more sensitive to a variety of apoptotic stimuli. The pro-apoptotic activity of Gal-7 was attributed to the activation of the JNK pathway in cervical cancer [8,9]. Zhu and colleagues confirmed these results by revealing a role for Gal-7 in sensitizing cervical cancer cells to paclitaxel treatment. Gal-7 knockdown in cancer cells shows increased viability against paclitaxel-induced apoptosis [10]. Analysis transcriptome of the HeLa cells that re-expressed Gal-7, multiple gene modules and transcriptional regulators were modulated in response to Gal-7 reconstitution [11]. It is noted that some genes among those are related to lipid metabolic regulation. Hence, we speculated that Gal-7 may be involved in the regulation of lipid metabolism.

Dysregulation in lipid metabolism is among the most prominent metabolic alterations in cancer. Cancer cells harness lipid metabolism to obtain energy, components for biological membranes, and signaling molecules needed for proliferation, survival, invasion, metastasis, and response to the tumor microenvironment impact and cancer therapy [12]. Glycerol is seen in biological systems as an intermediate in lipid metabolism. As major components of glycolipids and phospholipids, fatty acids (FAs) can be esterified with a glycerol moiety to form triglycerides (TAG), which are nonpolar lipids synthesized and stored in lipid droplets during high nutrient availability and hydrolyzed to generate ATP by FA oxidation under energy stress conditions [13]. In recent years, glycerol has been reported to act as a chemical chaperone to correct the conformation of proteins that cause human diseases [14,15]. To date, the glycerol-loaded co-crystal structures of Gal-3 (PDB: 5NFC), Gal-4N (PDB: 5DUU), Gal-8N (PDB: 5GZC) and Gal-13 (PDB: 7CYB) have been reported [16,17,18,19]. The three carbon and three oxygen atoms of glycerol occupy similar positions as C4, C5, C6 and O4, O5, and O6 of galactose, which is the ligand of the galectin family. In addition, the ability of glycerol to bind with the carbohydrate-binding site varies under different crystallization conditions. Therefore, we speculate that Gal-7 may also bind glycerol and regulates glycerol metabolism in HeLa cells.

In the present study, the interaction between Gal-7 and glycerol was studied in vitro. Results showed that glycerol can improve the thermal stability of Gal-7 and inhibit Gal-7-mediated erythrocyte agglutination. Gal-7 was crystallized in complex with glycerol under two different conditions. Glycerol can bind a new pocket near the N-terminus in addition to the carbohydrate-recognition binding sites. Moreover, overexpression of Gal-7 in HeLa cells decreased intracellular triglyceride levels but increased mRNA expression of AQP-3. Overall, our results will provide further insights into the understanding of the function of human Gal-7.

## 2. Results

### 2.1. Glycerol Improves Stability of Gal-7

**Figure 1:**
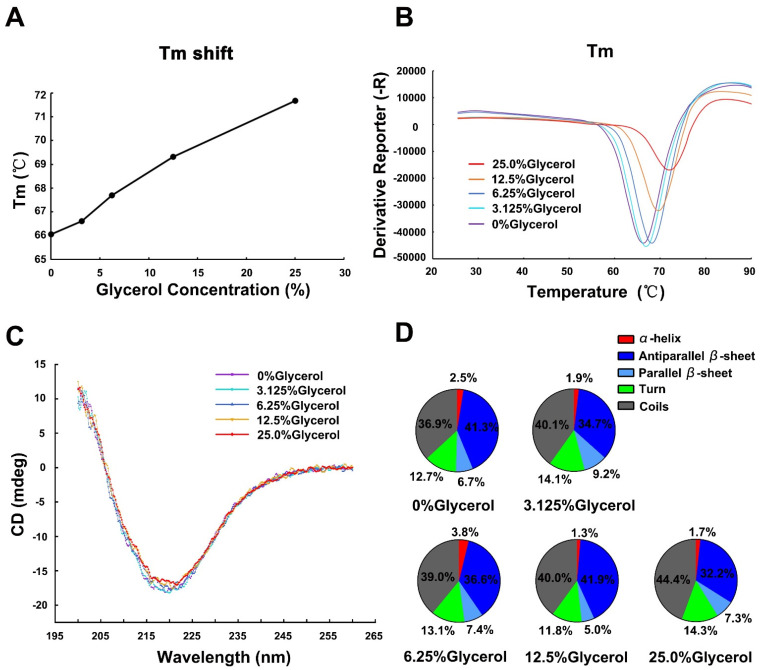
Thermal stability and secondary structural characteristics of Gal-7 in glycerol solutions. (**A**) Thermal shift assay. Tm shift when Gal-7 is incubated with different concentrations of glycerol. (**B**) Glycerol increases the melting temperature of Gal-7, indicating that glycerol can bind to Gal-7 and stabilize the structure of Gal-7. (**C**) CD assay. Profiles of CD spectrum of Gal-7 in glycerol solutions. The first negative derivative of the curve and CD spectrum of Gal-7 in 0%, 3.125%, 6.25%, 12.5%, and 25% glycerol are labeled purple, cyan, blue, yellow, and red, respectively. (**D**) Secondary structure content of human Gal-7 incubation with different concentrations of glycerol. The secondary structure content is shown as a percentage (%).

Compound binding usually helps to stabilize the protein due to protein-ligand interactions, resulting in an increase in the melting temperature (Tm) of the target protein during the thermal denaturation process [20]. We used the thermal shift assay to assess whether glycerol could influence the Tm of Gal-7. The Tm value of Gal-7 is 66.06 °C. However, the Tm value of Gal-7 is increased to 66.6 °C, 67.69 °C, 69.32 °C and 71.67 °C when 3.125%, 6.25%, 12.5% and 25.0% glycerol are present, respectively (Figure 1A,B). This finding indicates that glycerol can stabilize the structure of Gal-7.

### 2.2. Secondary Structure of Gal-7 in Glycerol Solutions

The secondary structure of Gal-7 in glycerol solution was measured by far-UV, as shown in Figure 1C. Taking into account the strong UV absorbance of the hydroxyl group of glycerol in the ultraviolet vacuum region (λ < 200 nm), the far-UV CD spectra were presented in a wavelength range from 200 to 260 nm. A typical β-sheet dominated spectra was observed with a broad negative band centered at 218 nm. Deconvolution was carried out using BeStSel.com. As shown in Figure 1D, the proportions of sheet structure are 48%, and the proportions of random coils are 36.9% when Gal-7 is without glycerol incubation. The proportions of sheet structure slightly decreased, while that of random coils slightly increased in the presence of glycerol. The far-UV CD spectra showed little difference at different glycerol concentrations, indicating that the secondary structure of Gal-7 was hardly influenced by the increase in glycerol concentration.

### 2.3. Hemagglutination Activity

**Figure 2:**
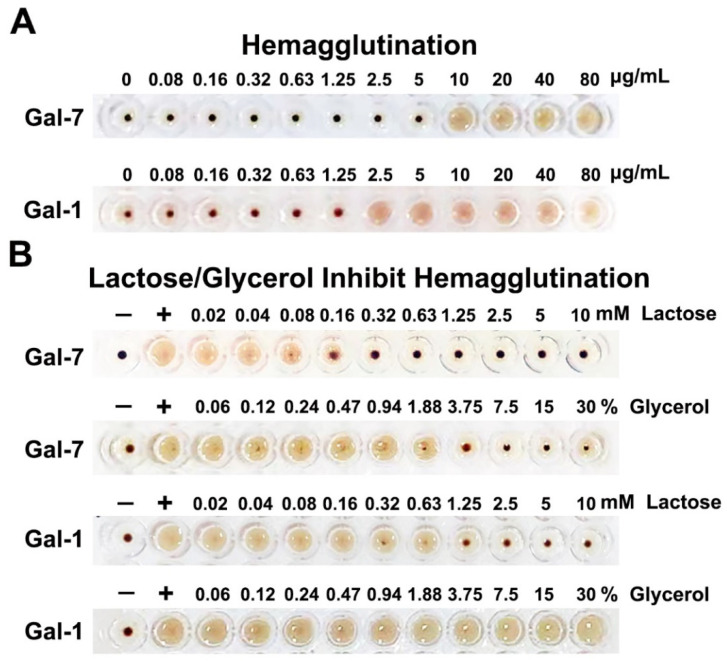
Hemagglutination assay. (**A**). Gal-7-induced agglutination of chicken erythrocytes with a MAC value of 10.0 μg/mL. However, the MAC value for Gal-1 was only 2.5 μg/mL. (**B**). Lactose could inhibit agglutination of chicken erythrocytes induced by Gal-7 and Gal-1. Glycerol could inhibit Gal-7-induced agglutination of chicken erythrocytes but could not inhibit Gal-1. The concentration of Gal-7 and Gal-1 used in the inhibition assays were 10.0 μg/mL and 2.5 μg/mL, respectively. ‘+’ represents the positive control containing Gal-7 or Gal-1 but no lactose or glycerol. ‘−’ represents the negative control of no Gal-7 or Gal-1.

The hemagglutination assay is a standard method to evaluate the inhibitory effects of ligands on galectins. The bioactivities of Gal-7 were assessed in the hemagglutination assay. We found that Gal-7 could promote chicken erythrocyte agglutination with a minimum agglutination concentration (MAC) of 10 μg/mL. We also determined the hemagglutination activity of Gal-1 as a control with a MAC value of 2.5 μg/mL (Figure 2A). The inhibitory effects of glycerol and lactose on Gal-7 and Gal-1 were also determined. Lactose is a very effective inhibitor of Gal-7-mediated chicken erythrocyte agglutination (MIC: 0.3125 mM), as well as Gal-1-mediated chicken erythrocyte agglutination (MIC: 1.25 mM). While 3.75% of glycerol was needed to inhibit Gal-7-induced hemagglutination of chicken erythrocytes, even 30% of glycerol could not inhibit Gal-1 (Figure 2B). This finding indicates that glycerol can bind to Gal-7, and glycerol is a ligand of Gal-7.

### 2.4. Crystal Structures of Gal-7 in Complex with Glycerol

**Figure 3:**
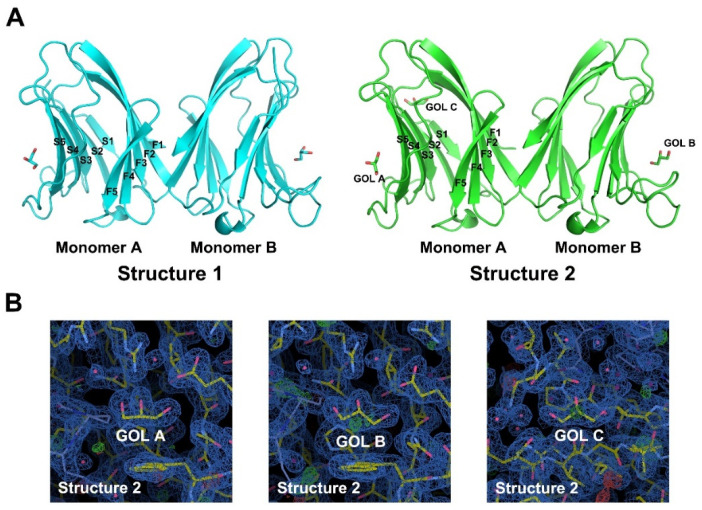
Structure of Gal-7 in complex with glycerol. (**A**) Gal-7 was crystallized as a dimer. In structure 1, each monomer can bind one glycerol; however, monomer A of structure 2 can bind two glycerols. (**B**) The electron density for the three glycerol molecules showed them to be different in their binding pocket. The 2|*Fo*|-|*Fc*| map contoured at 1.0 σ is shown in blue.

The two refined structures, structure 1 (homodimeric Gal-7 binds two glycerol molecules) and structure 2 (homodimeric Gal-7 binds three glycerol molecules), were crystallized using two different conditions, one at a relatively low concentration of PEG3350 (12%) and the other at a higher concentration (20%). As with all other galectins, structures 1 and 2 adopt the typical “jelly-roll” fold formed by sandwiching two anti-parallel β-sheets that consist of six (S1-S5) and five (F1–F5) β-strands and a short α-helix connecting strands S2 and F5 at 1.8 Å and 2.0 Å resolution, respectively. (Figure 3A). Our Gal-7 in complex glycerols as dimers in the P212121 space group is the same as previously reported for Gal-7 [21]. Structural statistics for Gal-7 in complex glycerols are provided in Table 1. Homodimeric Gal-7 binds three glycerol molecules at its two carbohydrate recognition binding sites (GOL A and GOL B) and the new binding pocket formed by its N-terminal and loop connecting strands S2 and F1 (GOL C), respectively. We found that electron density contour profiles for the three glycerol molecules were different in their crystal structure. Even though we were able to refine these glycerol molecules in the carbohydrate-binding sites and the binding pocket of the two monomers (Figure 3B), their different electron densities may be an effect that arises from crystal packing. The clear electron density map for the glycerol in its binding pocket reflects the molecular mechanism of Gal-7 binding to glycerol. The result of the co-crystal structure of Gal-7 and glycerol is the most likely direct evidence for the binding of Gal-7 to glycerol.

Differences in Cα RMSD values compared to Gal-7 structures (PDB: 1BKZ) are relatively small (RMSD 7XAC versus 1BKZ = 0.584 Å, RMSD 7XBL versus 1BKZ = 0.343 Å, RMSD 7XBL versus 7XAC = 0.685 Å). This indicates that the binding of glycerol hardly changes the overall structure of Gal-7, but changes may occur in certain regions.

**Figure 4:**
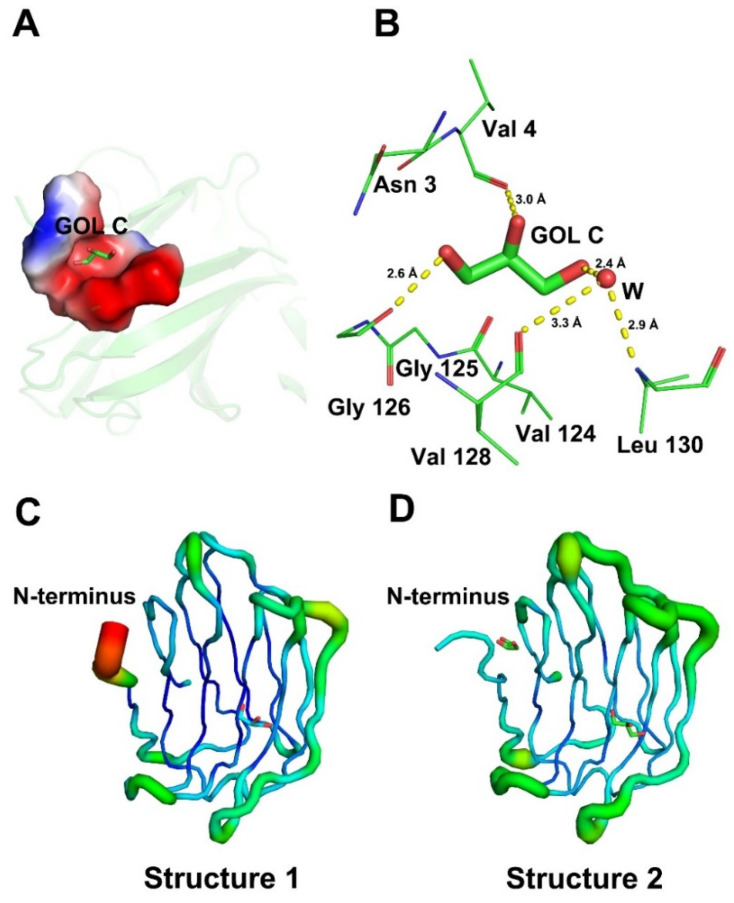
Glycerol-binding sites of Gal-7. (**A**) The region of the N-terminal (Asn 3-Val 4) and loop connecting strands S2 and F1(Val 124-Leu 130) formed a GOL C-binding pocket. (**B**) GOL C in its binding pocket is stabilized by Val 4, Gly 126 and a nearby water molecule through hydrogen bonds (colored yellow). Water molecule is labeled by deep salmon. (**C**) B-factor analysis of monomer A of structure 1. (**D**) B-factor analysis of monomer A of structure 2.

Monomer A of structure 2 can bind two glycerols. Its N-terminal (Asn 3-Val 4) and loop connecting strands S2 and F1(Val 124-Leu 130) form a GOL C-binding pocket (Figure 4A). GOL C can form hydrogen bonds with Val 4 and Gly 126, respectively. In addition, GOL C interacts indirectly with Val 128 and Leu 130 through a water molecule in the new ligand binding pocket of Gal-7. This means that the N-terminal of Gal-7 interacts with loop connecting strands S2 and F1 via a hydrogen bond bridge formed between GOL C and a molecule of water (Figure 4B). Discrepancies between structure 1 and structure 2 in monomer A are due to the absence or presence of GOL C. B-factors of structure 1 suggests that the N-terminal regions are very flexible, while the N-terminal region of structure 2 is relatively stable due to the binding of GOL C (Figure 4C,D). This indicates that the binding of glycerol hardly changes the overall structure of Gal-7, but changes occurred in certain regions that formed the GOL C-binding pocket.

### 2.5. Glycerol-Binding Sites of Gal-7

**Figure 5:**
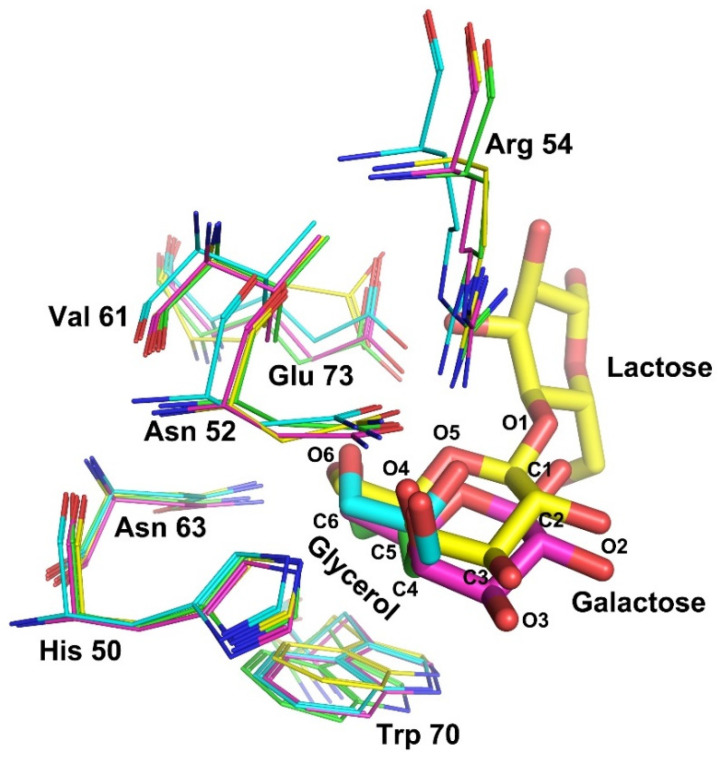
The carbohydrate binding sites of Gal-7 are merged. The residues of ligand-binding sites from structure 1, structure 2, and Gal-7 in complex with galactose (PDB: 2GAL) and Gal-7 in complex with lactose (PDB: 4GAL) are labeled by cyan, green, magenta and yellow, respectively. The positions of residues (His 50, Asn 52, Arg 54, Val 61, Asn 63, Trp 70 and Glu 73) could coordinate with glycerol and lactose. The C and O atoms of glycerol occupy similar sites as C4, C5, C6 and O4, O5, and O6 of galactose.

The carbohydrate binding sites of Gal-7 are highly conserved relative to other galectins. The positions of residues His 50, Asn 52, Arg 54, Val 61, Asn 63, Trp 70 and Glu 73 coordinate with glycerol. We superimposed the glycerol-binding sites of structures 1 and 2 with the galactose and lactose binding sites of Gal-7 (PDB: 2GAL and 4GAL). Except for the side chain orientation of Glu 73 in lactose-binding sites different from others, the positions and side chain orientations of the other ligand-binding sites are almost identical. This indicates that the presence of glycerol hardly influences the geometric parameters of the carbohydrate-binding sites of Gal-7. The Glu 73 residue has been suggested to coordinate the pyranose ring of lactose in the Gal-7 structure. It seems that this glutamic acid in Gal-7 can adopt different conformations depending upon which ligand binds to Gal-7. In our crystal structure, the C and O atoms of glycerol occupy similar positions as the C4, C5, C6 and O4, O5, and O6 atoms of galactose in the lactose-bound structure (Figure 5). It indicates that glycerol is a small ligand of Gal-7.

### 2.6. Overexpression of Gal-7 in HeLa Cells

**Figure 6:**
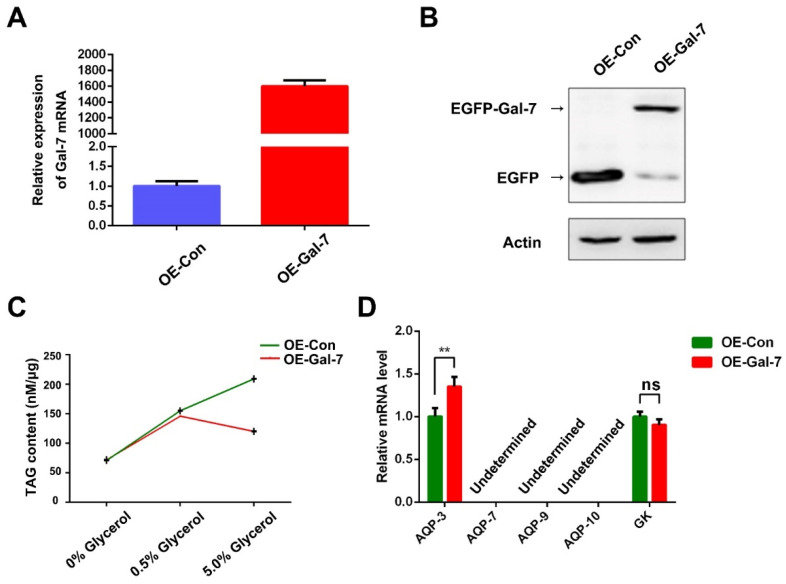
Overexpression of Gal-7 decreased the intracellular TAG levels but increased the mRNA expression of AQP-3 in HeLa cells. (**A**) mRNA expression of Gal-7 in HeLa by qRT-PCR. The Ct value of the Gal-7 gene was normalized to that of β-actin. Relative expression level was computed using 2^−ΔΔCt^ method. The data are represented as mean ± S.D. from three independent experiments. (**B**) Protein expression of Gal-7 in HeLa by Western blot. The level of β-actin was used as a control for equal amounts of input protein. Three independent experiments were performed, and one representative blot is shown. (**C**) The cellular TAG concentration was quantified by employing a TAG detection kit. Bars represent the mean ± SEM of three independent experiments. (**D**) The expression of AQP-3, AQP-7, AQP-9, AQP-10 and GK were assessed at 6 h after the stimulation with 5.0% glycerol. The Ct value of the target genes was normalized to that of β-actin. Relative expression level was computed using 2^−ΔΔCt^ method. Bars represent the mean ± SEM of three independent experiments. ** *p* < 0.01.

Here, overexpression of Gal-7 in HeLa cells (OE-Gal-7) was obtained by transient transfection. RT-PCR and Western blot analysis after transfection for 48 h showed that the mRNA level of Gal-7 in OE-Gal-7 cells was 1600-fold changed and showed a considerable increase in Gal-7 protein level (Figure 6A,B). Therefore, we have confirmed that Gal-7 was efficiently overexpressed in HeLa cells. To test the transfection efficiency, three batches of OE-Gal-7 cells in good condition were analyzed for Gal-7 protein levels by Western blot (Figure S1). The quantitative results of Gal-7 protein levels showed that there was no significant difference in Gal-7 expression among the three batches of OE-Gal-7 cells, indicating that the variation in transfection efficiency was not statistically significant. 

### 2.7. Overexpression of Gal-7 Decreased the Intracellular TAG Levels in HeLa Cells

Glycerol is mainly transported across the cytomembrane and is vital for lipid synthesis. OE-Gal-7 cells were grown in a medium with glycerol as a carbon source. Tumor cells need more energy than normal cells. TAGs are nonpolar lipid molecules composed of a glycerol molecule associated with three fatty acid (FA) molecules, and they represent the main form of lipid storage and energy in the human organism. We validated the correlation between Gal-7 expression and TAG levels in HeLa cells. As shown in Figure 6C, higher supplementary glycerol concentrations resulted in higher intracellular TAG levels. In addition, cellular TAG levels were obviously lower in OE-Gal-7 cells transfected with the pEGFP-N1-Gal-7 vector than in OE-Con cells when incubated with 5% glycerol. This indicates that overexpression of Gal-7 decreased the intracellular TAG levels in HeLa cells. 

### 2.8. Overexpression of Gal-7 Increased the mRNA Expression of AQP-3 in HeLa Cells

Glycerol can be the backbone of TAG. Several processes and many intermediate materials are implicated in TAG formation. AQPs are membrane proteins that act as channels for the transport of glycerol and other small solutes (including water, lactate and purines) across the membrane. Enzymes are needed in promoting the catalytic reaction chain to fulfill the TAG production. Several enzymes were involved in glycerol metabolism and lipid generation, such as glycerol kinase (GK), which turned glycerol into glycerol-3-phosphate (G3P) [22,23]. We have discovered that intracellular TAG is markedly reduced after overexpression of Gal-7; we next measured the AQPs and GK mRNA levels in HeLa cells to see whether Gal-7 is necessary for maintaining glycerol metabolism. As shown in Figure 6D, the AQP-3 levels in OE-Gal-7 cells were remarkably higher than the OE-Con cells but not GK and other AQPs. Increased AQP-3 levels favor cellular uptake of glycerol; however, the GK content is unchanged. It will cause glycerol molecules to accumulate in the cell and cause damage to the cells. According to these results, we speculated that overexpression of Gal-7 increased glycerol uptake but inhibited TAG formation. The accumulation of intracellular glycerol in OE-Gal-7 cells may cause cell damage and cause cells to be more sensitive to a variety of apoptotic stimuli.

## 3. Discussion

Glycerol is seen in biological systems as an intermediate in lipid metabolism. In recent years, glycerol has been reported to act as a chemical chaperone to correct the conformation of proteins. Ohnishi suggests that glycerol is effective in restoring several P53 mutants to normal P53 function [24]. In addition, glycerol can be used as a cryoprotectant in cryoannealing, a technique used to improve the quality of crystal data sets and minimize R values. After several cycles of flash-cooling and room temperature exposure, the quality of crystals is presumed to be better [25]. Currently, the glycerol-loaded co-crystal structures of Gal-3(PDB: 5NFC), Gal-4N (PDB: 5DUU), Gal-8N (PDB: 5GZC) and Gal-13(PDB: 7CYB) have been reported [16,17,18,19]. All glycerol binding is positioned at the carbohydrate-binding sites of galectins, which suggests that glycerol may act as a potential ligand. However, the functional and biochemical effects of glycerol on galectins are unknown. Gal-7; also termed p53-induced gene 1 product, belonging to prototype galectins, is a multifunctional effector by productive pairing with distinct glycoconjugates and protein counter-receptors in the cytoplasm and nucleus, as well as on the cell surface [4]. In this report, the binding of glycerol to Gal-7 improves stability and modulates its functions.

The interaction between Gal-7 and glycerol was studied in vitro. Results showed that glycerol can improve the thermal stability of Gal-7 and inhibit Gal-7-mediated erythrocyte agglutination. This indicates that glycerol acts as a ligand of lectin. For further study of the structural characterization of Gal-7 binding glycerol, circular dichroism (CD) was used to determine the secondary structure of Gal-7 in glycerol solutions. The results show that the secondary structure of Gal-7 was hardly influenced by the increase in glycerol concentration. In addition, Gal-7 was crystallized in complex with glycerol under two different conditions; one condition contained 20% PEG 3350, 0.2M magnesium chloride, and 0.1M HEPES, pH7.5, and the other condition included 0.1M HEPES, pH7.5, and 12% PEG 3350. It is noted that glycerol can bind a new pocket near the N-terminus in addition to the carbohydrate-recognition binding sites. N-terminal (Asn 3-Val 4) and loop connecting strands S2 and F1(Val 124-Leu 130) formed the new pocket, and glycerol can form hydrogen bonds with Val 4 and Gly 126, respectively. In addition, glycerol interacts indirectly with Val 128 and Leu 130 through a water molecule. Glycerol binds the pocket near the N-terminus of Gal-7, which can greatly reduce the flexibility and improve the stability of this region. It is noted that glycerol binds to GOL C sites of monomer A but not monomer B in structure 2. One possible reason to explain this phenomenon may be related to crystallization conditions. The structure of GOL C sites may vary under different crystallization conditions, which results in different abilities to bind glycerol. Just like Gal-7 structure 1 in the crystallization condition (20% PEG 3350, 0.2M magnesium chloride, 0.1M HEPES, pH7.5), no electron density of glycerol was found in GOL C sites, but glycerol electron density was found in GOL C sites in structure 2 of Gal-7 under another crystallization condition (0.1M HEPES, pH7.5, 12% PEG 3350). Another possibility may be related to the quality of the crystals. The quality of the monomer A part is good; therefore, enough diffraction data can be received by X-ray diffraction, while the quality of the monomer B part is not good enough. Therefore, the electron density map of glycerol is only found in monomer A but not monomer B. The third possibility is that the binding of monomer A to the glycerol molecule may inhibit the binding of monomer B to the glycerol molecule. Mayo and colleagues found the binding of one lactose molecule to a Gal-1 monomer A can negatively regulate the binding of the other [26]. At present, most inhibitors have been directed towards the carbohydrate-recognition binding sites, a challenging task in terms of specificity given the high structural homology of the carbohydrate-recognition binding sites among galectins [27]. The new glycerol binding pocket sites may act as a new drug target for designing specific inhibitors to Gal-7.

Our hemagglutination results show that glycerol could inhibit Gal-7-induced hemagglutination but not Gal-1. Gal-1 and Gal-7 belong to prototype galectin, contain one CRD per subunit and are noncovalently linked homodimers. Gal-1 dimers are formed via non-covalent interactions between the N- or C-terminal residues of two monomers. However, Gal-7 forms dimers via its F-face, with several residues forming hydrogen and ionic bonds, as well as van der Waals interactions that promote its dimeric structure (Figure S2A,B). In addition, the carbohydrate-binding sites of Gal-1 and Gal-7 are conserved, but there are differences in their structure and specificity for binding carbohydrate ligands. Mayo and colleagues found that the β-strands and loops around the carbohydrate-binding sites of Gal-1 are more open and dynamic in the unbound state [26]. This is confirmed by our alignment of the ligand-unbound Gal-1 structure (PDB ID: 2KM2) with the lactose-bound structure (PDB ID: 1GZW). In particular, the three amino acid (Arg 49, Trp 69 and Arg 74) side chains of Gal-1 that play a very important role in binding ligands show different orientations. However, the β-strands and loops around the carbohydrate-binding sites of Gal-7 were conservative whether binding glycerol or not (Figure S2C,D). The open and dynamic structure of Gal-1 may impair the ability to bind small molecule glycerol. In addition, we found the GOL C pocket, which binds glycerol in structure 2 of Gal-7, is deep and negatively charged with surrounding amino acids. However, the GOL C pockets of ligand-unbound Gal-1 and lactose-bound Gal-1 are smaller and shallower than Gal-7. Amino acids near the GOL C pockets of ligand-unbound Gal-1 and lactose-bound Gal-1 are mostly neutral and positively charged (Figure S2E–G). All of the above may cause differences in Gal-1 and Gal-7 binding to glycerol. Glycerol binds at the carbohydrate-recognition binding sites of Gal-7 based on our crystallization finding as well as lactose binding sites to Gal-7 (Figure 5). The results of hemagglutination assay with the lactose and glycerol added in different order showed that both lactose and glycerol inhibit Gal-7-induced agglutination by binding to the carbohydrate recognition sites of Gal-7, which is consistent with the crystallographic findings (Figure S3).

Gal-7 participates in diverse cancer processes [5]. It is reported that Gal-7 is negatively regulated in cervical cancer. Increased Gal-7 expression has been shown as a positive predictive biomarker for clinical responses after adjuvant radiation therapy in cervical cancer patients [7]. Ectopic expression of Gal-7 in HeLa cells renders the cells more sensitive to a variety of apoptotic stimuli [8]. Juan Carlos Higareda-Almaraz and colleagues examined the re-expression of Gal-7 in HeLa cells, analyzed their transcriptome, and found multiple genes and transcriptional regulators involved in lipid metabolic regulation were modulated in response to Gal-7 reconstitution [11]. It is well known that dysregulation in lipid metabolism is among the most prominent metabolic alterations in cancer [12].The glycerol component of triglycerides can be converted into glucose via gluconeogenesis to serve as a source of energy for the body. Glycerol is seen as an intermediate in lipid metabolism. We speculate that Gal-7 may regulate the level of lipid metabolism in HeLa cells. We validated the correlation between Gal-7 expression and TAG levels in HeLa cells. TAG levels were obviously lower in OE-Gal-7 cells than in OE-Con cells. This indicates that overexpression of Gal-7 decreased the intracellular TAG levels in HeLa cells. We analyzed gene expression associated with glycerol uptake and found the AQP-3 level of OE-Gal-7 cells was remarkably higher than the control cells but not GK and other AQPs. Increased AQP-3 levels favor cellular uptake of glycerol; however, the GK content is unchanged. OE-Gal-7 cells increase glycerol uptake, but glycerol conversion to TAG was inhibited. It will cause glycerol molecules to accumulate in the cells. One possibility is that excess glycerol molecules in cells may firmly bind Gal-7 and cannot be free; the other possibility is that the excess glycerol molecules in cells may increase osmotic pressure and cause harm to cell survival. All these can inhibit the conversion of glycerol to TAG. Therefore, ectopic expression of Gal-7 in HeLa cells may render the cells more sensitive to a variety of apoptotic stimuli by increasing glycerol uptake and inhibiting the conversion of glycerol to TAG.

## 4. Materials and Methods

### 4.1. Cell Lines and Culture Conditions

HeLa cells and Gal-7-transfected HeLa cells were maintained in Dulbecco’s modified Eagle’s medium (DMEM; Gibco, Rockville, MD, USA) supplemented with 10% fetal bovine serum (FBS), 0.1 unit/mL penicillin-streptomycin and 5% CO_2_.

### 4.2. Cloning, Overexpression, and Purification of Gal-7

The method of cloning, protein expression, and purification has been described in our earlier paper with some modifications [28]. PCR products of Gal-7 (residues 1–136, UniProt code: P47929) containing NdeI and XhoI restriction sites were cloned into pET28a. The constructs were transformed into BL21 pLysS cells (Tiangen Biotech) and grown in LB medium supplemented with kanamycin (100 μg/mL) and chloramphenicol (20 μg/mL). When the optical density of the cultures reached 0.6–0.8, IPTG was added to a final concentration of 0.5 mM to induce protein expression. After 16 h of induction at 25 °C, the cells were harvested by centrifugation and lysed by sonification in a lysis buffer consisting of 10 mM Tris/HCl, pH 7.5, 150 mM NaCl and 20 mM imidazole. The clarified cell extract was used for protein purification with Ni-NTA Agarose (Qiagen). After purification, the His-tagged protein was dialyzed in 10 mM Tris/HCl, pH 7.5, and 150 mM NaCl. During dialysis, thrombin was added to remove the His-tag with 3 units (National Institutes of Health unit) per milligram protein. As determined by sodium dodecyl sulfate/polyacrylamide gel electrophoresis (SDS/PAGE), protein purity was >90%. Finally, the protein was concentrated to 10 mg/mL using an Amicon Ultra-15 Centrifugal Filter Unit (3 kDa cutoff) and stored at −80 °C.

### 4.3. Thermal Shift Assay

The method of the thermal shift assay has been described in our earlier paper with some modifications [29]. In brief, the thermal shift assay was performed using the ABI StepOne/StepOnePlus Real-Time Detection System (ABI, Foster City, CA, USA). For this, 15 μg of Gal-7, 0.3 μL of 250 × SyPRO orange, 6 μL of 5 × PBS and the different concentrations of glycerol (0, 3.125, 6.25, 12.5, 25%) were added to wells in the 96-well PCR plate (BIOplastics, Landgraaf, The Netherlands). The plate was heated from 25 °C to 95 °C at a heating rate of 4 °C/min. The fluorescence intensity was measured with Ex/Em = 490/530 nm.

### 4.4. Circular Dichroism

Circular dichroism (CD) (JASCO J-815, Jasco Co., Tokyo, Japan) was used to determine the secondary structure of Gal-7 in glycerol solutions. Gal-7 was diluted by glycerol solutions at a concentration of 0.1 mg/mL for far-UV (200–260 nm) measurements, and cuvettes with a 1 mm path length were used. The samples were equilibrated at 4 °C for 2 h before measurements. The scanning was performed in a continuous mode of 50 nm/min. Glycerol solutions were taken as background and subtracted. All measurements were carried out in duplicates. The quantification of secondary structure was analyzed by the deconvolution software Bestsel to compare the percentages of secondary structure [30].

### 4.5. Hemagglutination Assay

The hemagglutination assay was performed according to a previous procedure with some modifications [28,29,31]. Chicken erythrocytes were prepared from fresh blood collected in Alsever’s medium (2.05% glucose, 0.8% sodium citrate, 0.42% sodium chloride, and 0.055% citric acid) and were washed four times with 0.15 M NaCl. Cells were then suspended in 4% (*v*/*v*) PBS (pH 7.4) containing 1 mg/mL trypsin and incubated at 37 °C for 1 h. After washing with 0.15 M NaCl, the cells were fixed for 1 h in PBS (pH 7.4) containing 1% glutaraldehyde at room temperature, followed by termination with five volumes of 0.1 M glycine in PBS (pH 7.4). The fixed cells were washed and adjusted to 10% (*v*/*v*) with PBS (pH 7.4). Cells were maintained at 4 °C until use. The hemagglutination assay was performed in microtiter V plates, with each well containing Gal-7 in 75 μL Tris buffer (10 mM Tris/HCl, 150 mM NaCl, pH 7.5) or 75 μL Tris buffer and 25 μL 4% (*v*/*v*) chicken erythrocyte suspensions. Cells were added last, followed by shaking, and agglutination was allowed to proceed for 60 min on the ice to ensure the temperature was consistent. Lactose and glycerol were used to inhibit the biological activity of Gal-7. The hemagglutination activity of Gal-1 and the inhibitory effects of glycerol and lactose on Gal-1 were also determined to compare with Gal-7.

### 4.6. Crystallization, Data Collection, and Structure Determination

Hampton Research packs (PEGRx1, PEGRx2, Index, Salt1, Salt2, Crystal Screen, and Crystal Screen 2) were used for the initial crystallization screen (sitting-drop vapor diffusion method). To obtain crystals suitable for X-ray diffraction, we first optimized crystallization using the common hanging-drop method. The final concentration of Gal-7 was 10 mg/mL. Gal-7 crystals appeared under two conditions. One condition (Index No. 84) containing 25% PEG 3350, 0.2M magnesium chloride, and 0.1M HEPES, pH7.5, and another condition (PEGRx1 No. 30) containing 0.1M HEPES, pH7.5, and 12% PEG 3350. To obtain a crystal suitable for X-ray diffraction, we optimized the crystal conditions and concentration of Gal-7. We were able to produce larger crystals from the two different conditions in approximately four days. One condition contained 20% PEG 3350, 0.2M magnesium chloride, and 0.1M HEPES, pH7.5. and the other condition included 0.1M HEPES, pH7.5, and 12% PEG 3350, which was similar to the initial condition; however, the Gal-7 concentration was 5.0 mg/mL to obtain the co-crystal structure of Gal-7 bound to glycerol. Prior to X-ray data collection, these crystals were soaked for approximately 1 min in 20% (*v*/*v*) glycerol and then flash-cooled in liquid nitrogen.

Data sets were indexed, integrated and scaled using HKL2000 [32]. Structures were determined by Phaser [33] with a molecular replacement method using the structure of Gal-7 (PDB:1BKZ) [21] as the search model. Structure refinement and water updating were performed using Phenix refine and manual adjustment [34,35]. Final structure validations were performed using MolProbity. Figures for all structures were generated using PyMOL or Coot [36,37].

### 4.7. Transient Transfection and Overexpression of Gal-7 in HeLa Cells

HeLa cells were plated in a 6-well plate 24 h prior to transfection and then were transiently transfected with pEGFP-N1-Gal-7 recombinant plasmids using the Lipo8000 transfection reagent, according to the manufacturer’s protocol (Beyotime). Controls were generated using HeLa cells transfected with the pECFP-N1 vector.

### 4.8. RT-PCR Analysis of Gal-7 mRNA in HeLa Cells

After transfection, the cells were plated in 6-well plates and cultured for 48 h. The cells were carefully washed with PBS, total RNA was isolated with TRIzol (Invitrogen), and cDNA synthesis was performed using 1 μg of total RNA with the ImpromII reverse transcription system (Promega). The primer sequences for Gal-7 were 5′-CCATGTAAACCTGCTGTGCG-3′ (forward) and 5′-GTTGAAGACCACCTCCGACG-3′ (reverse). The primer sequences for β-actin were 5′-CTGGAACGGTGAAGGTGACA-3′ (forward) and 5′-AAGGGACTTCCTGTAACAACGCA-3′ (reverse). Quantitative real-time PCR was performed with SYBR Green (Takara, Dalian, China), and the data collection was performed on the StepOnePlus Real-Time PCR Systems according to the manufacturer’s instructions. The relative expression level of the Gal-7 gene was compared with that of β-actin, and expression fold changes were calculated using 2^−ΔΔCt^ methods. The threshold cycle (CT) is the cycle at which the fluorescence level reaches a certain amount (the threshold). ΔCT = CT(Gal-7 gene)−CT(β-actin gene), and ΔΔCT = ΔCT(OE-Gal-7 sample)−ΔCT(OE-Control sample). All procedures were conducted according to the manufacturer’s guidelines. Experiments were performed in triplicate for each data point.

### 4.9. Western Blot

Western blot analysis was carried out using methodology from a previous study [31]. The primary antibodies for EGFP-tag and β-actin, as well as HRP-conjugated goat anti-mouse IgGs, were purchased from Huabio-antibodies, and the signals were detected using an enhanced chemiluminescence (ECL) plus kit (Yeasen Biotechnology) and FluorChem E system (ProteinSimple) according to the manufacturer’s directions.

### 4.10. Cellular TAG Measurement

After transfection, the cells were plated in 6-well plates and cultured for 48 h. The cells were carefully washed with PBS and maintained in Dulbecco’s modified Eagle’s medium supplemented with different concentrations of glycerol (0, 0.5 and 5.0%) without glucose and FBS for 6 h. The cells were collected and lysed by lysis buffer containing 1% Triton X-100, 50 mM Tris-HCl, pH 8.0, 150 mM NaCl and 1 mM PMSF. The cellular TAG level was determined using a TAG detection kit (NanJing JianCheng Bioengineering Institute) according to the manufacturer’s protocol. TAG levels of different groups were normalized to cell protein amount. Three independent experiments were performed in triplicate.

### 4.11. RT-PCR Analysis of AQPs and GK in HeLa Cells

After transfection, the cells were plated in 6-well plates and cultured for 48 h. The cells were carefully washed with PBS and maintained in Dulbecco’s modified Eagle’s medium supplemented with concentrations of 5.0% glycerol without glucose and FBS for 6 h. Total RNA was isolated with TRIzol (Invitrogen), and cDNA synthesis was performed using 1 μg of total RNA with the ImpromII reverse transcription system (Promega). The primers used were as follows: AQP-3, 5′-GGAATAGTTTTTGGGCTGTA-3′ (forward) and 5′-GGCTGTGCCTATGAACTGGT-3′ (reverse); AQP-7, 5′-GAGGAAGATGGTGCGAGAGTTC-3′ (forward) and 5′-CAAGTTGACACCAAGGTAGCTCC-3′ (reverse); AQP-9, 5′-ACTGCTGATCGTGGGAGAAAA-3′ (forward) and 5′-GCGTTCGCCAGAGATAGATACG-3′ (reverse); AQP-10, 5′-TCTCTGGCCGTTACGATAGC-3′ (forward) and 5′-CGTCCAACGATGCACATGG-3′ (reverse); GK, 5′-CTGGGACAAGATAACTGGAGAGC-3′ (forward) and 5′-TCAACGGTAGACTGGGTTCTTA-3′ (reverse); β-actin, 5′-CTGGAACGGTGAAGGTGACA-3′ (forward) and 5′-AAGGGACTTCCTGTAACAACGCA-3′ (reverse). Quantitative real-time PCR was performed with SYBR Green (Takara, Dalian, China), and the data collection was performed on the StepOnePlus Real-Time PCR Systems according to the manufacturer’s instructions. The relative expression level of indicated genes was compared with that of β-actin, and expression fold changes were calculated using 2^−ΔΔCt^ methods. The threshold cycle (CT) is the cycle at which the fluorescence level reaches a certain amount (the threshold). ΔCT = CT(a target gene)−CT(β-actin gene) and ΔΔCT = ΔCT(OE-Gal-7 sample)−ΔCT(OE-Control sample). All procedures were conducted according to the manufacturer’s guidelines. Experiments were performed in triplicate for each data point.

## Figures and Tables

**Table 1:** Data collection and refinement statistics.

PDB Code	7XAC	7XBC
Resolution (Å)	19.85–1.80(1.84–1.80)	20.93–2.00(2.05–2.00)
Space group	P212121	P212121
Unit cell parameters (a, b, c) (Å), (α, β, γ) (°)	(53.60,64.98,71.16) (90.00,90.00,90.00)	(52.85,65.01,73.03) (90.00,90.00,90.00)
No. of measured reflections	24,115 (1388)	18,725 (1255)
No. of unique reflections	23,618 (1388)	17,567 (1255)
Completeness (%)	99.7 (99.3)	99.5 (97.9)
Multiplicity	1.0 (1.0)	1.1 (1.0)
<I/δ(I)>	18.6 (6.3)	31.2 (26.9)
Rmodel (%)	20.12	16.89
Rfree (%)	24.74	22.22
Rmsd bond lengths (Å)	0.01	0.01
Rmsd bond angles (°)	1.09	1.02
Ramachandran plotf residues infavored regions (%)	96.62	98.49
Ramachandran outliers (%)	0	0
ligands	Two glycerol	Three glycerol

## Supplementary Materials

The following supporting information can be downloaded at: https://www.mdpi.com/article/10.3390/ijms232012318/s1.
