# Supplementary material for: Binding of Glycerol to Human Galectin-7 Expands Stability and Modulates Its Functions
Source: Int J Mol Sci. 2022 Oct 14;23(20):12318. doi: 10.3390/ijms232012318 (PMC9604435; doi:10.3390/ijms232012318)
Supplement: Supplementary file 1 [file ijms-23-12318-s001.zip › ijms-1897362-supplementary.pdf]

Supplementary Figures

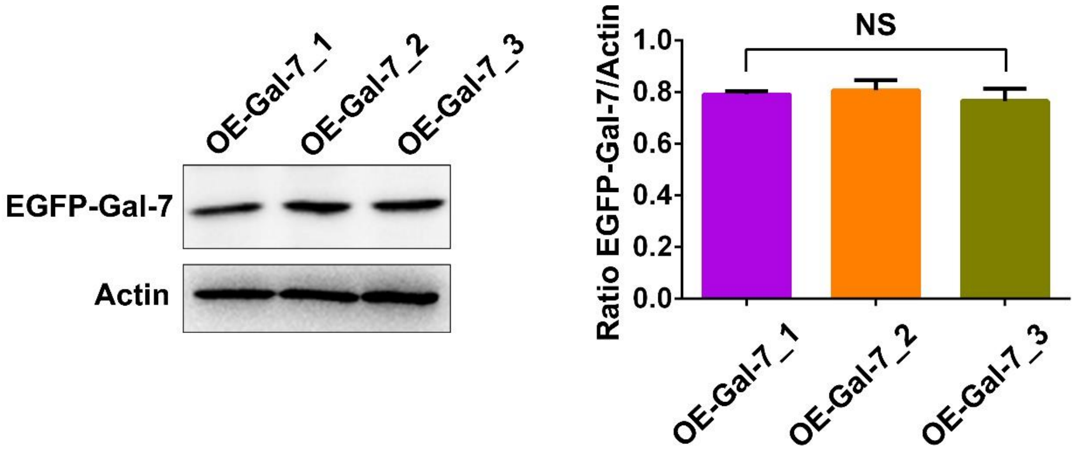

Figure S1. Western blot analysis of Gal-7 protein levels in three batches of OE-Gal-7 cells. Western blot using anti-EGFP antibody. The histogram to the right shows a quantification of EGFP-Gal-7 protein expressed as a ratio to actin. Values are no significantly different P=0.4461 > 0.05.

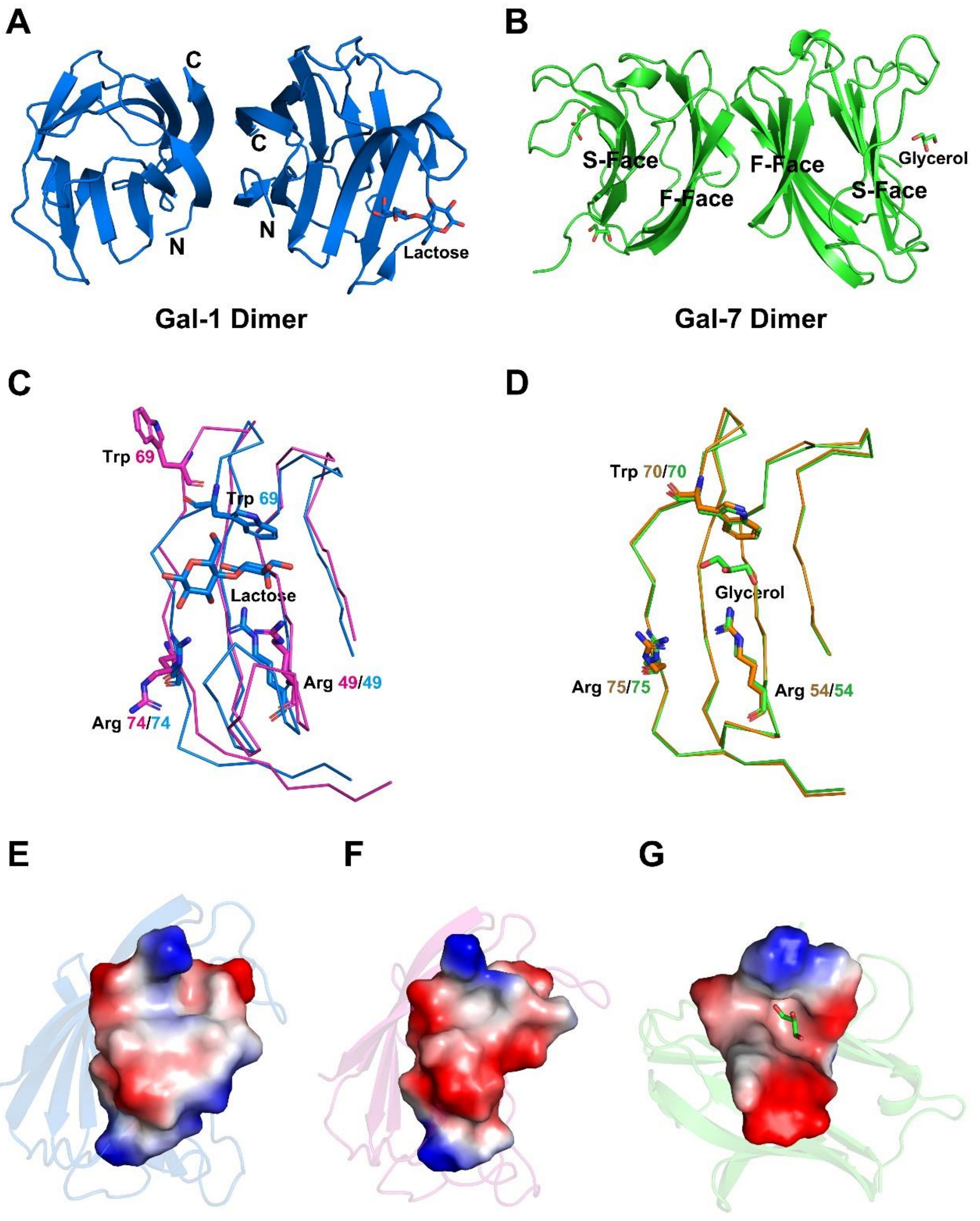

Figure S2. Structure of Gal-1 and Gal-7. (A) Structure of Gal-1 dimer binding lactose. (B) Structure of Gal-7 dimer binding glycerol. (C) Superposition of the structure around the carbohydrate binding sites between ligand-unbound Gal-1 (marine) and lactose-bound Gal-1(magenta). (D) Superposition of the structure around the carbohydrate binding sites between ligand-unbound Gal-7 (orange) and glycerol-bound Gal-7 (green). (E) Electrostatic potential surface of GOL C pockets in lactose-bound Gal-1. (F) Electrostatic potential surface of GOL C pockets in ligand-unbound Gal-1. (G) Electrostatic potential surface of GOL C pockets in glycerol-bound Gal-7.

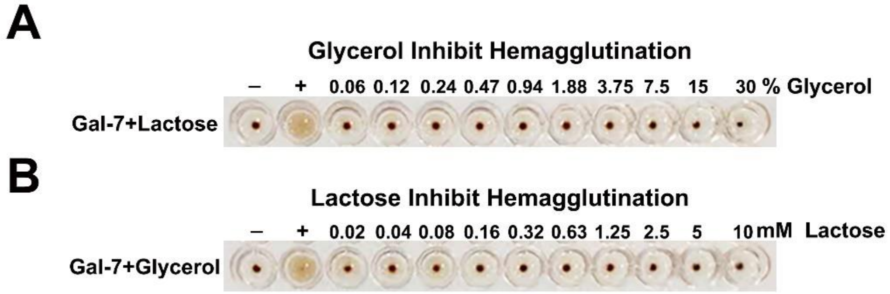

Figure S3. Hemagglutination assay. (A) 10 ug/mL Gal-7 and 0.3125 mM lactose were incubated on ice for 10 minutes, and then added to wells with different concentrations of glycerol to detect the inhibitory effect of glycerol on Gal-7-induced agglutination. Glycerol does not affect the inhibitory effect of lactose on Gal-7-induced agglutination even 30% of glycerol could not. (B) 10 ug/mL Gal-7 and 3.75% of glycerol were incubated on ice for 10 minutes, and then added to wells with different concentrations of lactose to detect the inhibitory effect of lactose on Gal-7-induced agglutination. Lactose does not affect the inhibitory effect of glycerol on Gal-7-induced agglutination even 10mM lactose could not. '+' represents the positive control containing Gal-7 but no lactose or glycerol. '-' represents the negative control of no Gal-7".
